# Red-Backed Vole Brain Promotes Highly Efficient *In Vitro* Amplification of Abnormal Prion Protein from Macaque and Human Brains Infected with Variant Creutzfeldt-Jakob Disease Agent

**DOI:** 10.1371/journal.pone.0078710

**Published:** 2013-10-24

**Authors:** Julie Nemecek, Nabanita Nag, Christina M. Carlson, Jay R. Schneider, Dennis M. Heisey, Christopher J. Johnson, David M. Asher, Luisa Gregori

**Affiliations:** 1 Laboratory of Bacterial and TSE Agents, Division of Emerging Transfusion-Transmitted Diseases, Office of Blood Research and Review, Center for Biologics Evaluation and Research, Food and Drug Administration, Rockville, Maryland, United States of America; 2 United States Geological Survey, National Wildlife Health Center, Madison, Wisconsin, United States of America; 3 Program in Cellular and Molecular Biology, University of Wisconsin, Madison, Wisconsin, United States of America; University of the Witwatersrand, South Africa

## Abstract

Rapid antemortem tests to detect individuals with transmissible spongiform encephalopathies (TSE) would contribute to public health. We investigated a technique known as protein misfolding cyclic amplification (PMCA) to amplify abnormal prion protein (PrP^TSE^) from highly diluted variant Creutzfeldt-Jakob disease (vCJD)-infected human and macaque brain homogenates, seeking to improve the rapid detection of PrP^TSE^ in tissues and blood. Macaque vCJD PrP^TSE^ did not amplify using normal macaque brain homogenate as substrate (intraspecies PMCA). Next, we tested interspecies PMCA with normal brain homogenate of the southern red-backed vole (RBV), a close relative of the bank vole, seeded with macaque vCJD PrP^TSE^. The RBV has a natural polymorphism at residue 170 of the PrP-encoding gene (N/N, S/S, and S/N). We investigated the effect of this polymorphism on amplification of human and macaque vCJD PrP^TSE^. Meadow vole brain (170N/N PrP genotype) was also included in the panel of substrates tested. Both humans and macaques have the same 170S/S PrP genotype. Macaque PrP^TSE^ was best amplified with RBV 170S/S brain, although 170N/N and 170S/N were also competent substrates, while meadow vole brain was a poor substrate. In contrast, human PrP^TSE^ demonstrated a striking narrow selectivity for PMCA substrate and was successfully amplified only with RBV 170S/S brain. These observations suggest that macaque PrP^TSE^ was more permissive than human PrP^TSE^ in selecting the competent RBV substrate. RBV 170S/S brain was used to assess the sensitivity of PMCA with PrP^TSE^ from brains of humans and macaques with vCJD. PrP^TSE^ signals were reproducibly detected by Western blot in dilutions through 10^-12^ of vCJD-infected 10% brain homogenates. This is the first report showing PrP^TSE^ from vCJD-infected human and macaque brains efficiently amplified with RBV brain as the substrate. Based on our estimates, PMCA showed a sensitivity that might be sufficient to detect PrP^TSE^ in vCJD-infected human and macaque blood.

## Introduction

Transmissible spongiform encephalopathies (TSEs), also known as prion diseases, are incurable and inevitably fatal neurodegenerative infections with long asymptomatic incubation periods. Variant Creutzfeldt-Jakob disease (vCJD) is a human TSE, causally linked to dietary exposure to bovine spongiform encephalopathy (BSE) agents [1,2]. Three clinical cases of transfusion-transmitted vCJD (TTvCJD) and one asymptomatic infection were reported in the U.K. All transmissions followed transfusions of non-leukoreduced red blood cells from donors who later became ill with vCJD [3-6]. One additional presumptive case of vCJD was reported in a patient with hemophilia and a long history of treatment with U.K.-sourced human plasma-derived factor VIII [7]. The number of food-borne vCJD cases reported worldwide each year is in decline; however, vCJD prevalence in the U.K. is still uncertain. A recent survey of appendix tissue in U.K. estimated that as many as 1 in 2,000 people had accumulation of abnormal prion protein PrP^TSE^ in lymphoid follicles [8]. These individuals are currently asymptomatic but might be incubating vCJD, if the presence of PrP^TSE^ implies vCJD infection; they may either remain in a silent carrier state and never develop clinical vCJD, or eventually progress to have overt vCJD. In any case, it seems prudent to consider blood of such individuals to be potentially infectious until proven otherwise [9]. Thus, despite the decline in the number of food-borne vCJD cases, TTvCJD remains a concern for public health. 

Currently, no vCJD test identifies blood donors with asymptomatic vCJD infections. A vCJD screening test suitable for detecting donors latently infected with the vCJD agent would improve the safety of blood transfusion and might also provide an estimate of true vCJD infection prevalence. All candidate antemortem blood-based tests currently under development are designed to detect PrP^TSE^ as the most reliable disease marker. PrP^TSE^ is presumed to be present in blood as it is in other infected tissues [10-12]. Estimates from measured infectivity titers of blood in rodents infected with TSE indicated that PrP^TSE^ concentrations might be in the order of only a few femtograms per mL of blood [13,14]. A recent analysis suggested that the levels of vCJD infectivity in human blood might be even lower than those in rodents [15]. If these estimates are correct, then direct detection of PrP^TSE^ in infected blood will be very challenging. One option is to increase the concentration of PrP^TSE^ in a blood sample to a detectable level with *in vitro* conversion of normal prion protein (PrP^c^) triggered by PrP^TSE^ to yield more PrP^TSE^. This type of technique has amplified trace amounts of PrP^TSE^ present in starting materials to levels detectable by Western blot or other immunological methods [12,16,17]. Other approaches to detect PrP^TSE^ with highly sensitive assay systems or specific antibodies are also being developed [10,12]. In the end, some of these candidate tests might mature into suitable vCJD blood screening tests, such tests will require validation using coded samples of known vCJD-infected and non-infected blood specimens. Validations should be conducted under experimental conditions that closely mimic the final intended screening assay platform. 

Methods that amplify PrP^TSE^ such as protein misfolding cyclic amplification (PMCA) and quaking-induced conversion (QuIC), have demonstrated extremely high sensitivity [11,16,18-20]. Both amplification protocols consist of mixing a small amount of PrP^TSE^ (the seed) from infected materials with large excess of PrP^c^ (the substrate) from uninfected sources. Usually, though not always, amplification is more efficient when the sources of the PrP^TSE^ seed and of the PrP^c^ substrate are matched: either materials from the same species or having PrP with the same aminoacid sequence (intraspecies PMCA). That suggests that PrP sequence homology between seed and substrate may favor conversion of PrP^c^ to PrP^TSE^. Both *in vitro* methods have been reported to amplify PrP^TSE^ in blood of experimentally infected rodents and sheep [20–22]. Promising as these preliminary studies with animal models may be, if PMCA or QuIC are to become successful assays suitable for vCJD blood donor screening, they must demonstrate a robust and efficient amplification of PrP^TSE^ in human blood. Because vCJD-infected human blood is not available in quantities sufficient to support assay development, we proposed to substitute blood of macaques experimentally infected with vCJD for blood of humans. Panels of macaque blood are currently being prepared in our laboratory to assist in vCJD assay validation (manuscript in preparation). In this work, we used PrP^TSE^ from vCJD-infected brain homogenates from macaques and humans to develop a highly efficient PMCA protocol. We estimated that the amplification of PrP^TSE^ achieved by our PMCA might offer the sensitivity necessary to detect PrP^TSE^ in naturally infected human blood. 

### Ethics Statement

This study was carried out in strict accordance with the recommendations in the Guide for the Care and Use of Laboratory Animals of the U.S. National Institutes of Health. The protocols were approved by the Institutional Animal Care and Use Committee of FDA CBER (ASP#2009-14). Voles were bred under a protocol approved by the Animal Care and Use Committee of the USGS National Wildlife Health Center (#EP080715-A2). 

Animal use was carried out in accordance with the Guide for the Care and Use of Laboratory Animals (8th Ed.) and the US Animal Welfare Regulations, with experiments approved by the FDA CBER IACUC. Welfare of caged nonhuman primates is enriched by a variety of approved methods, including social interaction, toys, novel food items and sensory stimulation (videos). The cages are manufactured by Primate Products built by Rockville Steel. Two cages are stacked and each cage has 6.0 sq ft of floor space and all cages have side shuttle doors for easy transfer of the animals from cage to cage. Each cage has a perch, 2 floor toys, foraging board and mirror. Animals are given enrichment every day. Animals also receive fruit daily and give trail mix on foraging board at least three days per week. A challenger basket is provided with treats once a week. Animals are under the care of trained technical staff if an animal required medical attention, a veterinarian determines the final course of action. Animals are monitored twice a day by the animal technical staff and a veterinarian is on call 24 hours a day 7 days per week. The FDA CBER-managed animal program is fully accredited by AAALAC International.

Macaques were euthanized with IV injection of Euthasol 100mg/kg.

The human brain material was purchased from the UK National Institute for Biological Standards and Controls (NIBSC). No IRB or ethics committee was required. Human tissue was anonymized. FDA laboratory is approved to work with human and macaque tissue and blood (HPRT#4867).

## Materials and Methods

### Preparation of brain tissues

Frozen brains of southern red-backed voles (*Myodes gapperi*) with all three naturally-occurring genotypes and meadow voles (*Microtus pennsylvanicus*) were obtained from the Prion Research Laboratory, USGS National Wildlife Health Center, Madison, WI. Human vCJD brain homogenate, WHO Candidate Biological Reference Materials, was obtained from the National Institute for Biological Standards and Control CJD Resource Centre UK [23,24]. Hamster brain homogenates uninfected and infected with the 263K strain of scrapie agent and macaque brain homogenates vCJD-infected and uninfected were obtained in-house. 

### Normal brain homogenization for PMCA

Particular care was taken to chill all materials and reagents overnight at 4°C. Fifty mL of homogenization buffer contained: 0.5 g lab-grade Triton X100 (Sigma), 1.5 mL of 5M molecular grade NaCl, and 48 mL of 1x phosphate-buffered saline (PBS) without calcium and magnesium, pH 7.2. Immediately prior to homogenization of brain, one protease inhibitor cocktail tablet containing EDTA (Roche) was added to 50 mL of homogenization buffer. Typically, 1 g of frozen brain (perfused with saline unless otherwise stated) was transferred to a chilled homogenizer (Kontes 15 mL tissue grinder, #885301-0015 or similar) and cold homogenization buffer was added to prepare a 10% w/v brain suspension. The tissue was homogenized immediately with 40 strokes with pestle A and then 60 strokes using pestle B, keeping the grinder on ice throughout. The homogenate was aliquoted into pre-chilled eppendorf tubes and snap-frozen in dry ice. Aliquots were stored at -80°C and thawed only once on ice following by thorough vortexing before using in PMCA. Separate homogenizers were used for each tissue. The homogenizers were rinsed in 2 N NaOH followed by distilled water after use.

### PMCA procedure

All PMCA studies were independently repeated by two investigators in the lab at different times. We used a Q700 sonicator (QSonica) fitted with a cup horn probe and a round tube rack. The sonicator water bath was filled with 300 mL water, and the water was changed after every experiment. The temperature was maintained by placing the cup horn on the appropriate stand in an incubator with temperature adjusted to 37°C. In a typical PMCA reaction, automated sonicator settings for one round of PMCA were: 20 sec sonication, 30 min incubation, 48 cycles (16-min processing time). Prior to preparing samples for PMCA, the work area in the biosafety cabinet was thoroughly cleaned with 2 N NaOH and rinsed with water. To control cross contamination, we handled uninfected and infected materials in separate areas and always used separate pipettors with filtered pipette tips. The operator donned a clean gown and double gloves that were changed frequently. Between rounds of PMCA, samples were stored at -80°C; samples were sonicated on the day of PMCA for 20 sec. before 1:10 dilution for the next round of PMCA. 

All infected brain homogenates were prepared as 10% w/v suspensions in PBS. Tissue homogenizations were conducted in a Minibeadbeater homogenizer (Biospec Products, Bartlesville, OK). Previous experience has demonstrated that this infectious brain homogenates may be frozen and thawed several times without reducing amplification efficiency. The seed and the substrate were mixed at the appropriate ratios, and a 100 µL aliquot of the mixture transferred to a flat-cap PCR tube (Fisherbrand, #14230225) along with three 3/32" PTFE beads (McMaster Carr, #9660K12). New beads were used for each round of PMCA. At least 2 negative controls were always included in every PMCA experiment and passaged serially along with experimental samples. PMCA reactions were set up by diluting 10% infected seed 10-fold into 10% normal brain homogenate in 100 µL reaction volume. All 10-fold serial dilutions of the seed in the substrate used the same procedure. The tubes were placed in the center of the rack at a distance of 3 to 6.5 cm from the edge of the cup horn rack. Reaction tubes were incubated in the sonicator for 30 min before starting the automated sonicator cycles. The power setting for sonication was determined by the wattage. Power was adjusted to ~ 199-215 Watts for each PMCA. 

### Proteinase K digestion and Western blotting

Typically, we conducted proteinase K (PK) digestion immediately after the end of sonication and stored samples at -80°C until use. Alternatively, some samples were stored at -80°C and sonicated for 20 sec in the Q700 at 37°C prior to PK digestion. To each 10 μL aliquot, PK and Sarkosyl were added at 50 µg/mL and 0.05% final concentrations, respectively. Reactions with vole substrates were incubated at 45°C; all other reactions were incubated at 37°C. All PK reactions were incubated for 1 hr while shaking at 450 rpm in a thermomixer (Eppendorf). The reaction was stopped by the addition of 1 μL of PMSF (20 mg/0.5 ml ethanol) followed by NuPAGE sample buffer with reducing and heating at 98°C for 10 min with shaking at 450 rpm. Samples were stored overnight at -20°C if not immediately loaded on gels for Western blot. We noticed that, in some instances, if samples were stored for long periods of time after digestion or frozen and thawed repeatedly, prion protein antibody-positive aggregates with molecular weights higher than those of PrP^TSE^ were observed on Western blots. Proteins were separated on precast Bis-Tris 12% precast gels at 200V for 60-45 min followed by transfer to PVDF at 30 V for 1 hr. Membranes were blocked with TBST (10 mM Tris, pH 8.0, 150 mM NaCl, 0.1% Tween-20) in 5% dry nonfat milk for 1 hr at room temperature. Prion protein was detected using either 6D11 (for all voles PrP) or 3F4 (for hamster, macaque or human PrP) monoclonal antibodies (purchased from Research Foundation for Mental Hygiene, NYS Institute for Basic Research, NY); AP-labeled secondary antibodies were used to visualize prion protein with Immun-Star AP substrate (BioRad). 

## Results

### Intraspecies PMCA with macaque vCJD

**Figure 1:**
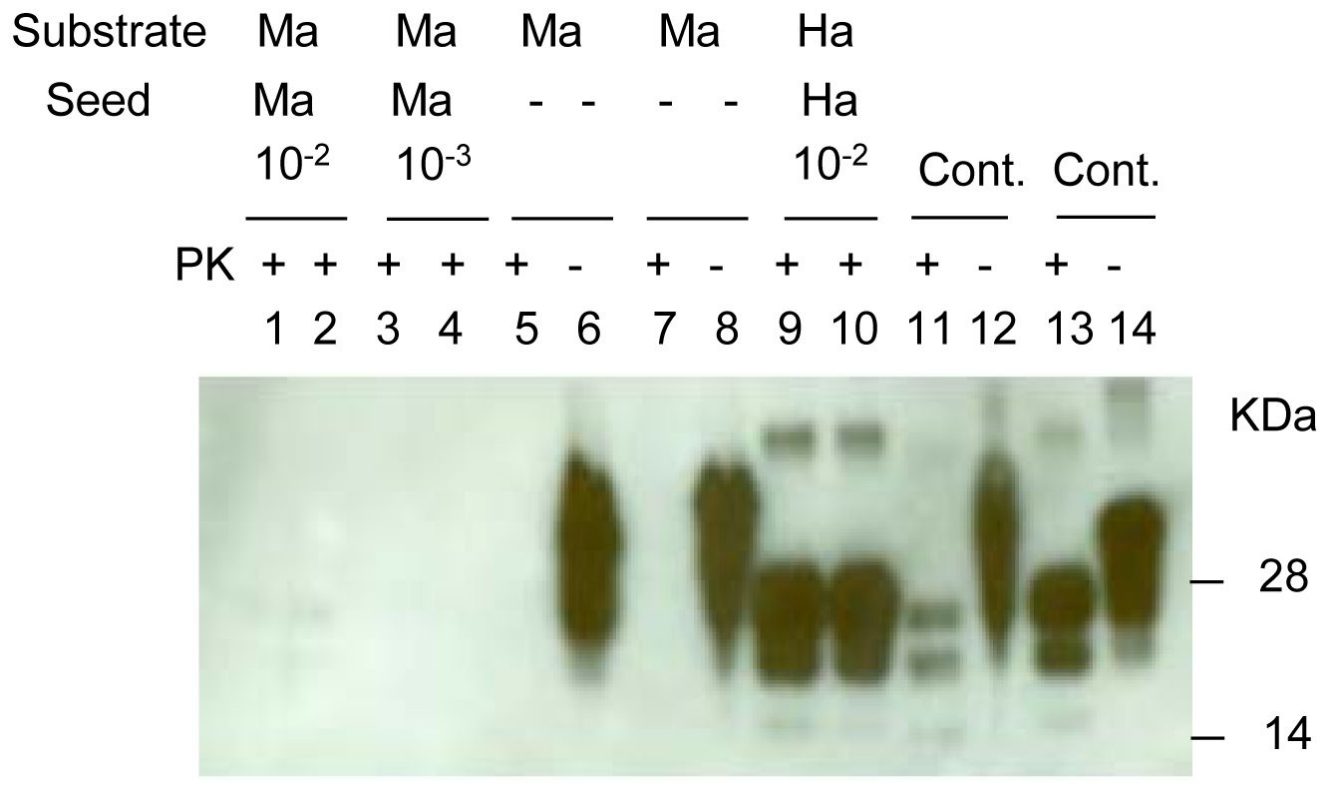
Intraspecies PMCA with vCJD macaque brain homogenate. A 10% suspension of brain tissue from macaque with terminal vCJD was diluted 10^-2^ or 10^-3^ into 10% normal macaque brain homogenate and subjected to 1 round of PMCA (lanes 1-4). Lanes 5-8 indicate 10% macaque NBH without seed subjected to 1 round of PMCA. A 10% brain homogenate from hamsters infected with scrapie (263K) was diluted 10^-2^ fold into 10% hamster NBH and subjected to 1 round of PMCA (lanes 9-10). Macaque vCJD brain homogenate (1%) was loaded in lanes 11-12 and 1% 263K scrapie hamster brain homogenate was loaded in lanes 13-14 as positive controls (Cont.). Prior to electrophoresis, samples were digested with proteinase K (PK) as indicated. The blot was stained with 3F4 antibody. Ma = macaque; Ha = hamster; PK = proteinase K.

We investigated the ability of PrP in normal macaque brain homogenate (substrate) to amplify PrP^TSE^ (seed) present in 10% vCJD-infected macaque brain suspension diluted 10^-2^ and 10^-3^ (Figure 1). All dilutions refer to 10% brain homogenate as zero dilution. As a control for PMCA, we used hamster intraspecies conversion with 10^-2^ dilution of 10% scrapie-infected hamster brain under the same PMCA conditions used with macaque brain. The results showed that with one round of PMCA (48 cycles) only intraspecies amplification with hamster brain seed and substrate produced detectable *de novo* PrP^TSE^ (lanes 9 and 10) while macaque brain substrate did not. We also repeated intraspecies macaque PMCA, varying the sonicator power settings and testing different PK digestion conditions with macaque PrP^TSE^ with no better outcomes (data not shown). 

### Interspecies PMCA with macaque vCJD PrP^TSE^ seed and vole brain substrate

**Figure 2:**
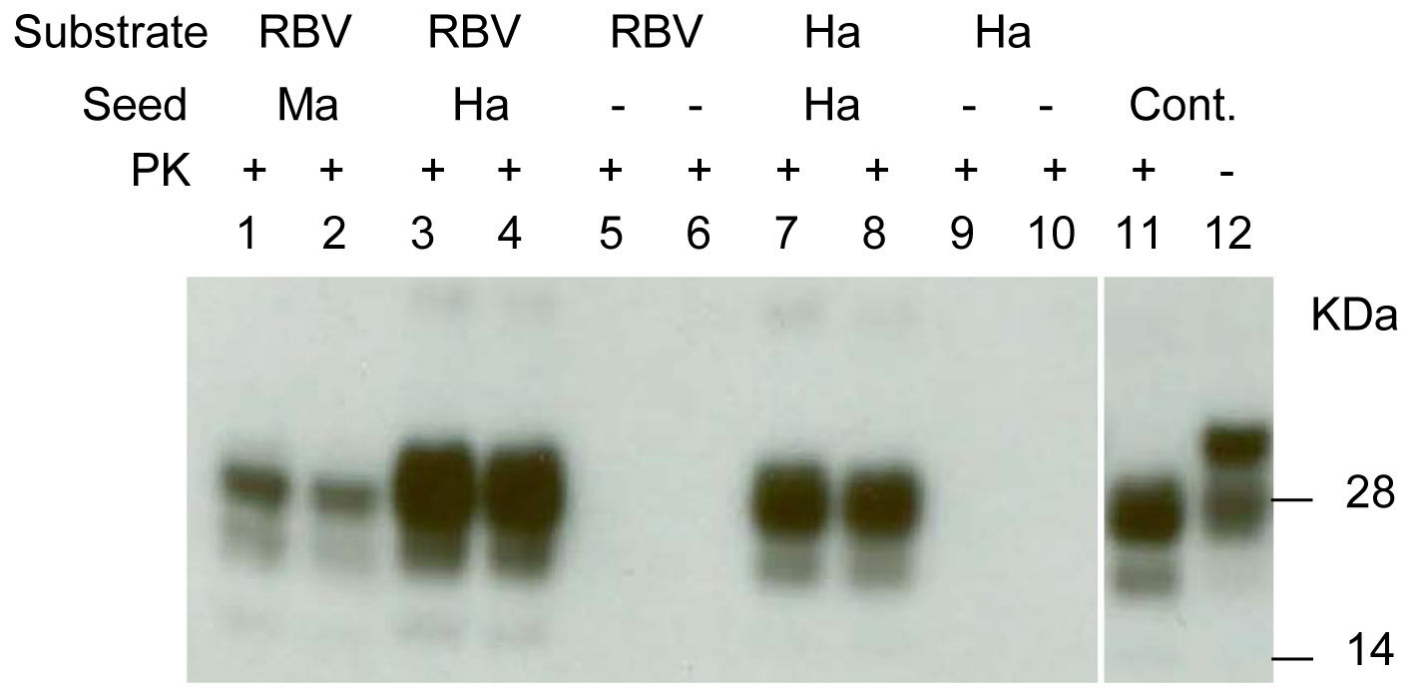
Interspecies PMCA with vCJD macaque brain homogenate and red-backed vole 170N/N as the substrate. Macaque vCJD brain material and hamster 263K scrapie brain material was diluted 10^2^-fold into 10% RBV 170N/N NBH and subjected to 1 round of PMCA (lanes 1-4 respectively). Lanes 5-6 indicate control PMCA samples containing 10% RBV 170N/N NBH substrate only without added seed. Scrapie hamster brain material was diluted 10^2^ fold into 10% hamster NBH and subjected to 1 round of PMCA (lanes 7-8). Lanes 9-10 indicate 10% hamster NBH without seed subjected to 1 round of PMCA. Scrapie hamster brain homogenate (1%) was loaded in lanes 11^-12^ as a positive control (Cont.). Prior to electrophoresis, samples were digested with PK. The blot was stained with 6D11 antibody.

Brain suspension of the Eurasian bank vole (*Myodes glareolus*) has been shown to be an excellent substrate for PMCA, amplifying PrP^TSE^ from various species including humans [25]. Bank voles are indigenous to Western Europe and Northern Asia but very difficult to obtain in the U.S. In this work we selected a close relative of the bank vole, the North American southern red-backed vole (RBV) as the substrate for PMCA with macaque and human PrP^TSE^ seeds. Figure 2 shows that RBV normal brain homogenate amplified PrP^TSE^ in brain homogenates from a vCJD-infected macaque (lanes 1 and 2) and a scrapie-infected hamster (lanes 3 and 4). The level of amplification of hamster PrP^TSE^ with vole substrate was similar, if not better, than that with hamster intraspecies PMCA (lanes 7 and 8). No PrP^TSE^ signal was detected when PMCA was conducted using either normal RBV or normal hamster brain homogenates without seed (lanes 5, 6, 9 and 10). 

### Effect of red-backed vole polymorphism at codon 170 of the PrP gene on PMCA efficiency

**Figure 3:**
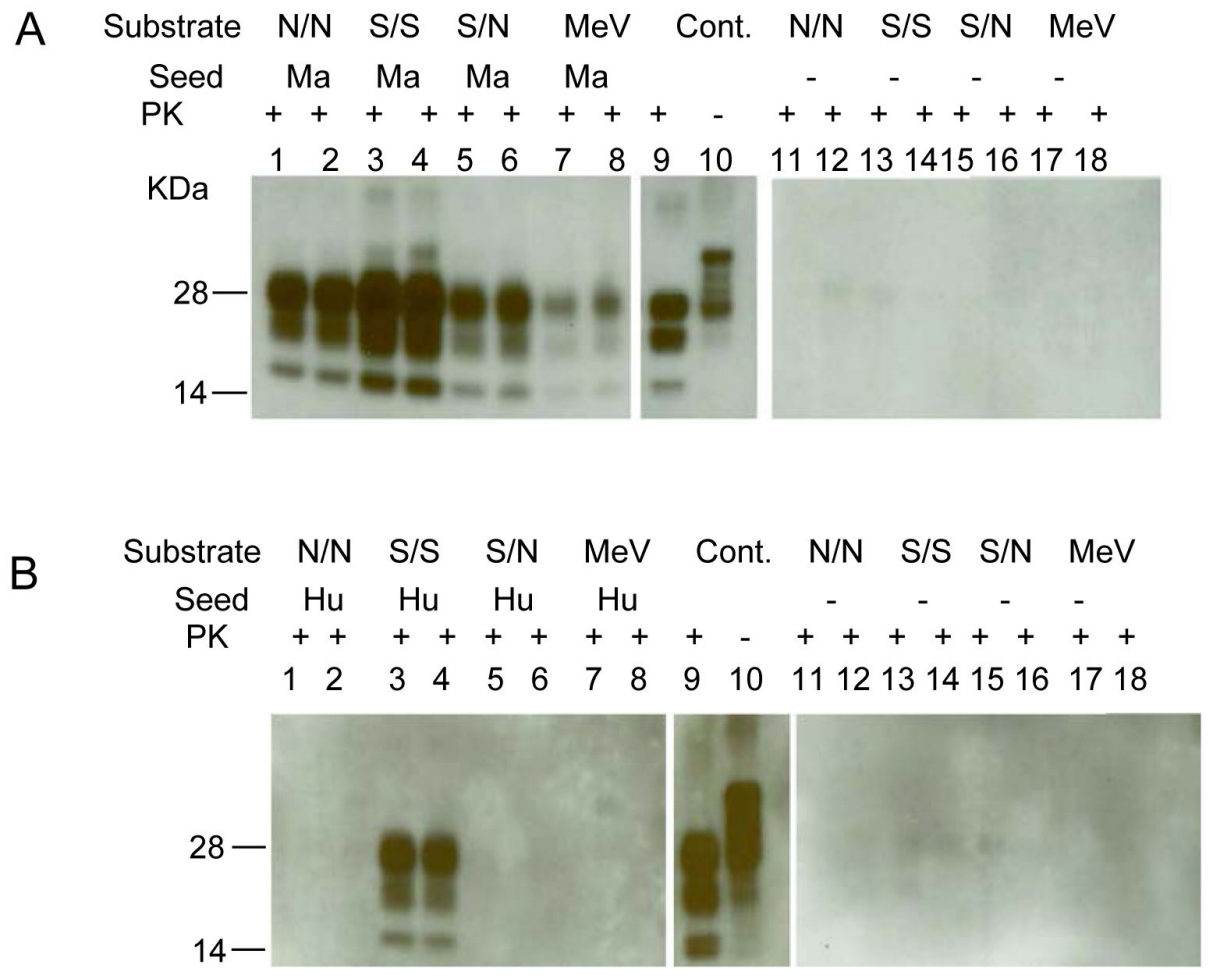
PMCA is affected by the red-backed vole PrP 170 polymorphism. (A) Brain material from macaques clinically ill with vCJD was diluted 10^-3^ into 10% RBV 170N/N NBH (lanes 1-2), into 10% RBV 170S/S NBH (lanes 3-4), into 10% RBV 170S/N NBH (lanes 5-6), or into 10% meadow vole (MeV) NBH (lanes 7-8) and subjected to 1 round of PMCA with 3 beads in each. Macaque vCJD brain homogenate (5%) was loaded in lanes 9-10 as positive control (Cont.). Lanes 11-18 indicate 10% RBV 170 (N/N, S/S, S/N respectively), MeV NBH without seed and subjected to 1 round of PMCA. Lanes 1-8 and 11-18 were stained with 6D11 antibody and lanes 9-10 were stained with 3F4 antibody. Prior to electrophoresis, samples were digested with PK. (B) Human vCJD brain material was diluted 10^-3^ into 10% RBV 170N/N NBH (lanes 1-2), into 10% RBV 170S/S NBH (lanes 3-4), into 10% RBV 170S/N NBH (lanes 5-6), into 10% meadow vole NBH (lanes 7-8) and subjected to 1 round of PMCA with 3 beads in each. Human vCJD brain homogenate (5%) was loaded in lanes 9-10 as positive control (Cont.). Lanes 11-18 indicate 10% RBV 170 (N/N, S/S, S/N), MeV NBH without seed and subjected to 1 round of PMCA. Prior to electrophoresis, samples were digested with PK. Lanes 1-8 and 11-18 were stained with 6D11 antibody and lanes 9-10 were stained with 3F4 antibody.

The RBV PrP gene (*PRNP*) gene has a natural polymorphism at codon 170, in the β2-α2 loop region of the prion protein supposedly important for both transmission of infectivity [26] and PMCA [27]. The predominant *PRNP* genotype in RBV is 170N/N; however, 170S/S and 170S/N genotypes also occur. Meadow voles, another vole species present in the U.S., are known to have only 170N/N PrP genotype. We tested brain homogenates prepared from RBV with the three genotypes and meadow voles to assess whether efficiency of PMCA correlated with prion protein aminoacid sequence similarity at position 170 between seed and substrate using either macaque PrP^TSE^ or human PrP^TSE^ as the seeds (Figure 3). Both humans and macaques have serine at PrP codon 170 with no known polymorphism. Table S1 shows the prion protein amino acid sequence alignment for all animal species used in this study. The results in Figure 3A indicate that macaque PrP^TSE^ was amplified by all brain homogenates tested; however, RBV 170S/S brain was the best substrate, and brain homogenate from meadow voles was a poor substrate compared to RBV. Using human PrP^TSE^ as the seed, PMCA was successful only with RBV S/S substrate, and all other substrates failed to support amplification (Figure 3B). 

### Effect of brain perfusion in PMCA

**Figure 4:**
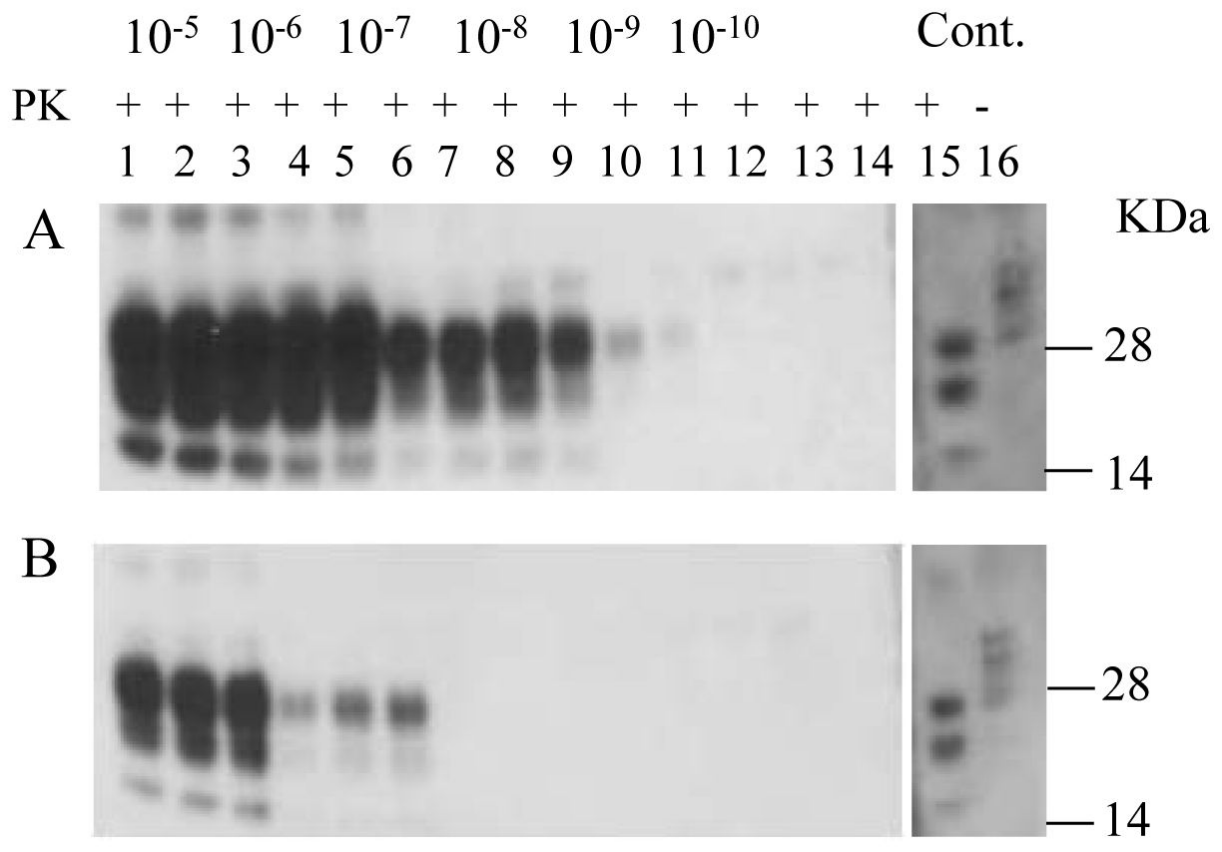
PMCA with perfused and non-perfused red-backed vole brains. Macaque vCJD brain material was diluted 10^-5^to 10^-10^ into a 10% RBV 170S/S perfused brain homogenate (Figure 4A), or into 10% RBV 170S/S non-perfused brain homogenate (Figure 4B) and subjected to 2 rounds of PMCA. PMCA samples without seed are shown in lanes 13 and 14 in both panels. PMCA results are shown in lanes 1-12 with saline-perfused RBV brains (panel A) and non-perfused RBV brains (panel B). Macaque vCJD brain homogenates (5%) with and without PK were loaded in lanes 15 and 16 in both panels as positive controls (Cont.). Prior to electrophoresis, samples were digested with PK. The blots were stained with 6D11 antibodies (lanes 1-14) and 3F4 antibodies (lanes 15-16).

Removal of blood from the brain by saline perfusion is considered necessary for optimal amplification of PrP^TSE^ by PMCA [19]. However, perfusion of a normal brain is time-consuming and, in some cases, impractical. In an effort to optimize PMCA for primate PrP^TSE^, we assessed the effect of perfusion of the brain used as substrate on the efficiency of PMCA. We compared the results with perfused and non-perfused RBV (170S/S) homogenates for amplification of macaque PrP^TSE^. Figure 4 shows an improvement in PrP^TSE^ amplification after one round of PMCA with perfused brains compared to non-perfused brains with seed diluted 10^-5^ to 10^-10^-fold. 

### Effect of beads in PMCA

**Figure 5:**
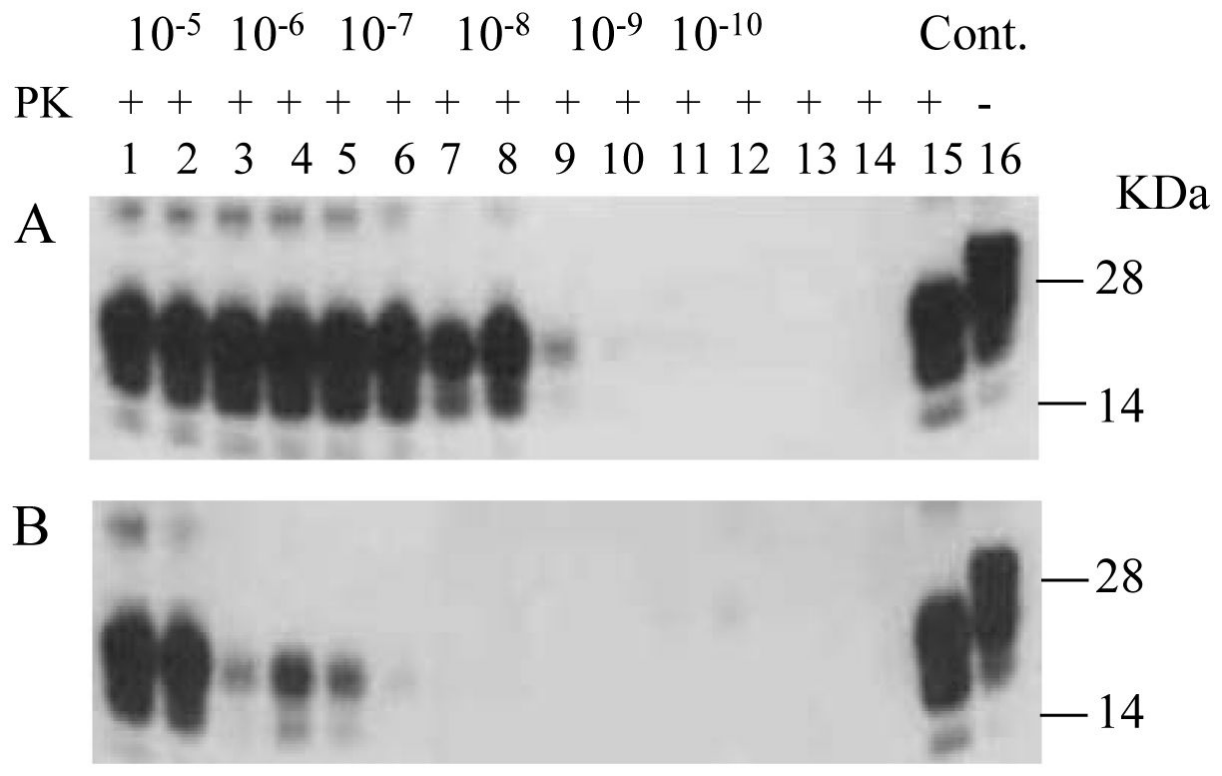
PMCA with and without beads in reaction tubes. Scrapie hamster (263K) brain material was diluted 10^-5^ to 10^-10^ in 10% hamster NBH and subjected to 1 round of PMCA with 3 beads added to each sample tube (Figure 5A) or without beads (Figure 5B). PMCA samples without seed are shown in lanes 13 and 14 in both panels. PMCA results are shown in lanes 1-12 with beads added (panel A) and without beads added (panel B). Scrapie hamster (263K) brain 1% homogenate was loaded in lanes 15-16 in both panels as positive controls (Cont.). Prior to electrophoresis, samples were digested with PK. The blots were stained with 6D11 antibodies.

We also investigated the effect of including beads in the PMCA reaction (Figure 5). In this study, we used hamster intraspecies PMCA starting with 10% scrapie-infected brain as PrP^TSE^ seed diluted 10^-5^ to 10^-10^ and 2 rounds of amplification. Three Teflon beads were added to appropriate tubes at the beginning of the PMCA procedure. We found increased PrP^TSE^ signals in the presence of beads and highly diluted seed (≥ 10^-6^-fold). It should be noted that beads were included in all other experiments described here. 

### PMCA with serially-diluted vCJD-infected macaque and human brain homogenates

**Figure 6:**
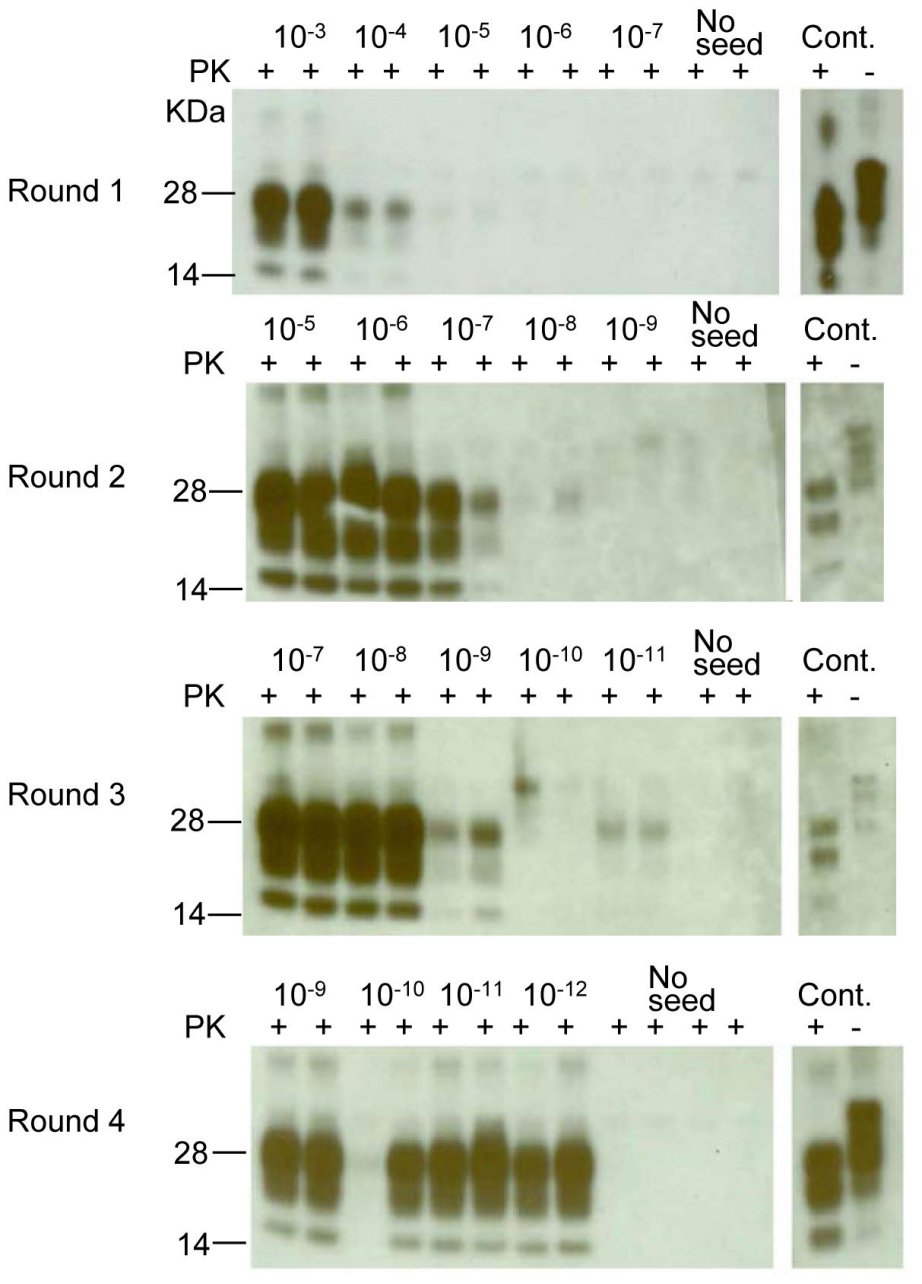
PMCA with a dilution series of vCJD macaque brain homogenate. Macaque vCJD brain was diluted 10^-3^-10^-12^ in 10% 170 RBV 170S/S saline-perfused NBH and subjected to 4 rounds of PMCA. Macaque vCJD 5% brain homogenate was loaded as positive control (Cont.) in all 4 rounds. Round 1 shows products of the 10^-3^-10^-7^ dilutions, Round 2 shows the 10^-5^-10^-9^ dilutions, Round 3 shows the products of 10^-7^-10^-11^ dilutions, Round 4 shows the products of 10^-9^-10^-12^ dilutions. Prior to electrophoresis, samples were digested with PK. All blots were stained with 6D11 antibody except the control lanes that were stained with 3F4 antibody.

Next we determined the sensitivity of PMCA with serial dilutions of 10% vCJD-infected macaque brain homogenate amplified with RBV 170S/S NBH as the substrate. The dilution range tested was 10^-3^ to 10^-12^ (relative to 10% brain homogenate). Each dilution was tested in duplicate. Results displayed in Figure 6 indicate that, in the first PMCA round, PrP^TSE^ generated from 10^-3^ and 10^-4^ dilutions of vCJD macaque brain homogenate were clearly detectable, and weak signals were visible with 10^-5^ dilution. In a second PMCA round, PrP^TSE^ was detected in dilutions 10^-5^ through 10^-7^, and in the third round, up to 10^-9^ dilution was detectable and both duplicates of 10^-11^ dilution showed weak signals, although no PrP^TSE^ was detected in the intermediate 10^-10^ dilution. In the fourth and last round of PMCA, 1 of the duplicate samples at 10^-10^ dilution contained barely detectable amounts of PrP^TSE^, while PrP^TSE^ was clearly visible all other dilutions through 10^-12^. The control duplicate samples with unseeded PMCA showed no PrP^TSE^, indicating that no obvious cross contamination had occurred. 

**Figure 7:**
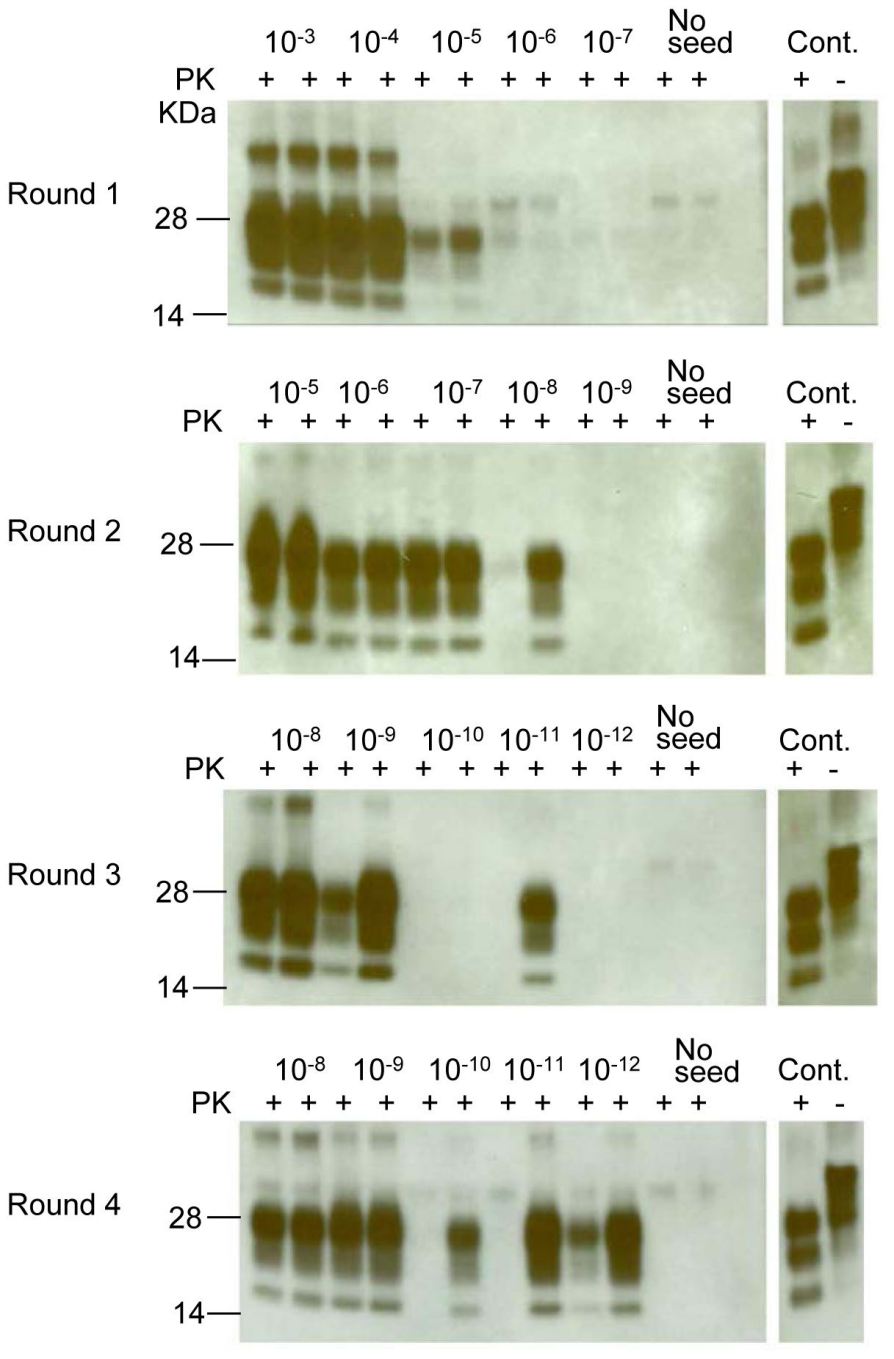
PMCA with dilution series of vCJD human brain homogenate. Human vCJD brain material was diluted 10^-3^-10^-12^ into 10% RBVS/S saline-perfused NBH and subjected to 4 rounds of PMCA. Human vCJD 5% brain homogenate was loaded as a positive control (Cont.) in all 4 rounds. Round 1 shows the products of 10^-3^-10^-7^ dilutions, Round 2 shows the products of 10^-5^-10^-9^ dilutions, Round 3 shows the products of 10^-8^-10^-12^ dilutions, Round 4 shows the products of 10^-8^-10^-12^ dilutions. Prior to electrophoresis, samples were digested with PK. All blots were stained with 6D11 antibody except the controls lanes that were stained with 3F4 antibody.

The same study was conducted with 10^-3^ to 10^-12^ dilutions of 10% vCJD-infected human brain homogenate as the seed and RBV 170S/S NBH as the substrate (Figure 7). Also in this case, after the first round of PMCA, PrP^TSE^ was detected in 10^-5^ dilution; in the second PMCA round, PrP^TSE^ was detected in both duplicates at 10^-7^ dilution and in one duplicate of 10^-8^ dilution. Third PMCA round detected PrP^TSE^ in both duplicates at 10^-8^ and 10^-9^ dilutions and in one duplicate at 10^-11^ dilution. The last PMCA round detected PrP^TSE^ in at least one duplicate of each dilution up to 10^-12^. No signal was detected in any control lanes with unseeded PMCA. 

## Discussion

We showed that RBV brain with 170S/S PrP genotype was an excellent substrate for *in vitro* amplification of PrP^TSE^ from human and macaque brains infected with the vCJD agent. Both primates have the PrP 170S/S genotype. Macaque PrP^TSE^ amplification was supported by all three RBV PrP genotypes, although RBV 170S/S was the most efficient substrate among the three. This non-strict requirement for matching of residue 170 in the seed and the substrate was also observed with PrP^TSE^ from brains of deer affected by chronic wasting disease amplified using RBV brains with the three genotypes as the substrates [28]. In striking contrast, amplification of human PrP^TSE^ absolutely required RBV 170S/S. Our data showed that only RBV 170S/S efficiently amplified both, human and macaque brains. However, macaque PrP^TSE^ appeared to be more permissive than human PrP^TSE^ and RBV 170N/N and 170S/N also supported its amplification. These data also suggest that the seed (PrP^TSE^ from macaques or humans) determined which RBV genotype would serve as a competent substrate for PrP^TSE^ amplification. 

To our knowledge, this is the first report showing interspecies PMCA amplification of human and macaque vCJD PrP^TSE^ seeded into RBV brain. Previously, intraspecies PMCA successfully amplified human sCJD PrP^TSE^ using as substrates human platelets, human brain homogenates or brains of transgenic “knock-in” mice expressing all three genotypes at 129 codon of the human *PRNP* gene [29-31]. In contrast, amplification of vCJD PrP^TSE^ required a substrate prepared from brains of mice expressing human 129M/M PrP [32]. Bank vole brains were shown to be very permissive PMCA substrates when seeded with brains infected with scrapie agent from various origins (sheep, hamster, mouse, and vole) and with brains from cattle with BSE and humans with sCJD [33]. Consistent with PMCA data, bank voles were also very susceptible to infections with a wide range of TSE agents, including human sCJD and scrapie of sheep and rodents, despite the low degrees of prion protein homology [33]. However, Agrimi noted that bank voles were relatively insensitive to infection with human vCJD agent [34]. It would be interesting to assess the efficiency of bank vole PrP, for which only 170N/N PrP genotype is known (Table S1), as substrate in the amplification of vCJD PrP^TSE^. 

We noted that sometimes the amplification signals in duplicate lanes were not identical suggesting different levels of efficiency. Usually, though not always, these differences were resolved in the subsequent round of PMCA. In other cases, PrP^TSE^ signals from higher dilutions were detected before signals from lower dilutions. We investigated these observations and excluded being caused by either the position of the tube in the sonicator or technical errors in dilutions. Most likely this phenomenon is due to the fact that PrP^TSE^ is aggregated and not homogenously disperse and duplicate samples of highly diluted PrP^TSE^ solutions could contain different amounts of the aggregated protein. We evaluated the effect of saline perfusion of normal brain and of addition of beads to the sample tubes in PMCA efficiency. We confirmed other investigators’ reports that these measures improve the level of amplification [19,20,35,36]. However, the effect was observed with low concentrations of the seed (≥ 10^-5^) and low number of PMCA rounds. We also showed that stringent use of aseptic-like conditions in our PMCA protocol ensured that all control samples containing no seed PrP^TSE^ remained negative even after four rounds of PMCA and dramatically lowered the rate of false positives. However, control of cross contamination in PMCA remains an important challenge [25]. 

One goal of this study was to assess whether PMCA offers the sensitivity needed to amplify PrP^TSE^ at concentrations close to those likely to be present in TSE infected blood. The concentration of PrP^TSE^ in infected blood or plasma is unknown. However, our earlier estimates, using the model of hamsters infected with 263K scrapie suggested a probable concentration of 3.2 x 10^-14^ g of PrP^TSE^ per mL of infected plasma [13]. We also estimated the probable concentration of PrP^TSE^ in 10% scrapie-infected hamster brain homogenate to be approximately 6 µg/mL [13]. These estimates are very close to those obtained by others using PMCA [14]. Assuming that these PrP^TSE^ concentrations estimated for the scrapie hamster model are similar to PrP^TSE^ concentrations in brain and in blood of humans infected with vCJD, then, a 10^-12^ dilution of 10% human brain homogenate should correspond to 6 x 10^-18^ g of PrP^TSE^ per mL. This initial concentration of PrP^TSE^ –a concentration more than 5,000-fold lower than the concentration of PrP^TSE^ estimated to be present in infected plasma (excess ratio = 3.2 x 10^-14^ g mL^-1^/6 x 10^-18^ g mL^-1^) was amplified to detectable levels after four rounds of PMCA. These data suggest that our PMCA might have sufficient sensitivity to detect PrP^TSE^ in infected human and monkey blood. These estimates are based on a set of assumptions derived from the well-characterized scrapie hamster model, assumptions that may or may not be the same for primates. However, we consider that if our assumptions have overestimated the concentration of PrP^TSE^ in primate brains, the amounts of PrP^TSE^ in 10^-12^ brain dilution would be lower and the ratio above would be more favorable for detection. Underestimation of PrP^TSE^ concentration is also possible, but we believe that to be unlikely, because our estimates of PrP^TSE^ concentration in scrapie-infected hamster brains (0.06 mg/g of brain) were relatively high [13]. The excess sensitivity (5000-fold) provided by our PMCA would serve to compensate for the anticipated inefficiency of PMCA conversion in blood compared to brain as well as probable lower titers of infectivity in primate blood compared with hamster blood. Blood and especially plasma are notoriously difficult assay matrices and represent a critical challenge to the development of a PMCA-based assay that will need to be addressed [19]. In conclusion, we have developed a highly sensitive PMCA capable of detecting extremely low concentrations of vCJD PrP^TSE^ in brain; the assay has the potential to serve as a proof-of-concept test to detect PrP^TSE^ in blood of humans and macaques infected with the vCJD agent. 

## Supporting Information

Table S1 **Prion protein amino acid sequence alignment of red-backed vole (*Myodes gapperi*), meadow vole (*Microtus pennsylvanicus*), bank vole (*Myodes glareolus*), golden Syrian hamster (*Mesocricetus auratus*), cynomolgus macaque (*Macaca fascicularis*), and human (*Homo sapiens*).** Amino acids at position 170, hypothesized to play a role in interspecies transmission and *in* *vitro* amplification of prion diseases, are highlighted. Red-backed voles are naturally polymorphic at residue 170, exhibiting the genotypes 170S/S, 170S/N, and 170N/N; only the 170S/S genotype is displayed in the figure for simplicity. The numbering convention follows the sequence for red-backed vole prion protein.
(DOC)
