# Supplementary material for: Red-Backed Vole Brain Promotes Highly Efficient In Vitro Amplification of Abnormal Prion Protein from Macaque and Human Brains Infected with Variant Creutzfeldt-Jakob Disease Agent
Source: PLoS One. 2013 Oct 24;8(10):e78710. doi: 10.1371/journal.pone.0078710 (PMC3813480; doi:10.1371/journal.pone.0078710)
Supplement: Table S1 — Prion protein amino acid sequence alignment of red-backed vole (Myodes gapperi), meadow vole (Microtus pennsylvanicus), bank vole (Myodes glareolus), golden Syrian hamster (Mesocricetus auratus), cynomolgus macaque (Macaca fascicularis), and human (Homo sapiens). Amino acids at position 170, hypothesized to play a role in interspecies transmission and in vitro amplification of prion diseases, are highlighted. Red-backed voles are naturally polymorphic at residue 170, exhibiting the genotypes 170S/S, 170S/N, and 170N/N; only the 170S/S genotype is displayed in the figure for simplicity. The numbering convention follows the sequence for red-backed vole prion protein. (DOC) [file pone.0078710.s001.doc]

Table S1. Amino acid sequences of all prion proteins used in this work

1 50

Red-backed vole MANLSYWLLAFFVATWTDVGLCKKRPKPGGWNTGGSRYPGQGSPGGNRYP

Meadow vole .............T....................................

Bank vole ..........L......N................................

Hamster ..........L.......................................

Macaque ....GC.M.VL.....S.L...............................

Human ....GC.M.VL.....S.L...............................

51 100

Red-backed vole PQGGGTWGQPHGGGWGQPHGGGWGQPHGGGWGQPHGGGWGQGGGTHNQWN

Meadow vole ..................................................

Bank vole .....-.................................S..........

Hamster ..................................................

Macaque .....G...........................................H

Human .....G........................................S...

101 150

Red-backed vole KPSKPKTNMKHVAGAAAAGAVVGGLGGYMLGSAMSRPMIHFGNDWEDRYY

Meadow vole ..................................................

Bank vole ..................................................

Hamster ..N....S...M..........................L...........

Macaque .......S...M.........................L......Y.....

Human ...........M................V........I....S.Y.....

151 170 200

Red-backed vole RENMNRYPNQVYYRPVDQY**S**NQNNFVHDCVNITIKQHTVTTTTKGENFTE

Meadow vole ...................**N**..............................

Bank vole ...................**N**..............................

Hamster ...................**N**..............................

Macaque ....Y..............**S**..............................

Human ....H..........M.E.**S**..............................

201 250

Red-backed vole TDVKMMERVVEQMCVTQYQKESQAYYEGRSSRAVLLFSSPPVILLISFLI

Meadow vole ................................................--

Bank vole .............................Y.H..VF.........L....

Hamster .............................R.-SAV...............

Macaque ..............I...E.......QRG..--MV...............

Human ..............I...ER......KRG..--MV...............

251 255

Red-backed vole FLIVG

Meadow vole -----

Bank vole .....

Hamster .....

Macaque .....

Human .....
